# OR7-003 – MEFV genotype, IL1B and role of NLRP3 in FMF

**DOI:** 10.1186/1546-0096-11-S1-A104

**Published:** 2013-11-08

**Authors:** A Omenetti, S Carta, L Delfino, A Martini, M Gattorno, A Rubartelli

**Affiliations:** 1 UO Pediatria II, G Gaslini IRCCS, Genoa, Italy; 2 University of Genoa, Genoa, Italy; 3 UO Cell Biology, IRCCS AOU San Martino - IST, Genoa, Italy

## Introduction

Familial Mediterranean Fever (FMF) is the most common of the hereditary autoinflammatory disorders. FMF is caused by mutations of *MEFV* gene which encodes for pyrin. It has been recently reported that frequency of FMF-like symptoms decreases from patients carrying two high penetrance mutations towards patients with a single low penetrance mutation. The effectiveness of interleukin (IL)-1b blockers has suggested that IL-1b may play a role in the pathophysiology of the disease. However, evidence of dysregulated IL-1b secretion in FMF patients is so far missing. Moreover, the role of NLRP3 has never been directly examined in FMF patients.

## Objectives

To define in patients affected by Familial Mediterranean Fever (FMF), whether or not interleukin (IL)-1β secretion (1) is enhanced, (2) correlates with the type of MEFV mutation and (3) is mediated by NLRP3.

## Methods

Freshly isolated monocytes from 20 FMF patients (12 homozygous and 8 heterozygous), 14 MEFV healthy carriers (HC) and 30 healthy donors (HD), unstimulated or after LPS-induced activation, were analyzed for redox state (reactive oxygen species (ROS) production and antioxidant responses), and for IL-1β and IL-1 Receptor antagonist (IL-1Ra) secretion. NLRP3 down-modulation was induced by NLRP3 *in vitro* silencing.

## Results

LPS-stimulated monocytes from FMF patients displayed enhanced IL-1β secretion which correlated with the number and penetrance of MEFV mutations. Silencing of NLRP3 consistently inhibited IL-1β secretion. As in other autoinflammatory diseases, MEFV mutated monocytes produced more ROS than genetically negative controls. However, contrary to CAPS, they were featured by a conserved and sustained antioxidant response. Consistent with this finding, MEFV mutated monocytes did not exhibit the functional indicators of oxidative stress observed in CAPS, including accelerated IL-1β secretion and deficient IL-1Ra production.

## Conclusion

MEFV mutated monocytes display enhanced IL-1β secretion which correlates with the number of high-penetrance mutations and level of endogenous ROS. Unlike NLRP3 mutated cells, monocytes carrying MEFV mutations withstand oxidative stress and preserve IL-1Ra production, thereby limiting inflammation. Finally, in contrast to what found in the animal model, the increased secretion of IL-1β by LPS-stimulated FMF monocytes is NLRP3-dependent.

## Disclosure of interest

None declared.

